# Wm. R. Warner & Co’s Pills

**Published:** 1874-08

**Authors:** 


### W. R. Warner & Co.'s Pills.

Recently an article has appeared ln the *American Journal of Pharmacy*, *The Detroit Review of Medicine and Pharmacy* and other medical journals giving an analysis of various samples of Quinine pills, named by number only some of which fell far short of the mark. At the request of Messrs. Wm. R. Warner & Co., of Philadelphia, Prof. A. R. Lyons, M. D., of Detroit, made an analysis of several of their pills selected from those for sale by their agent in Detroit. Prof. Lyons found their pills to be all that was claimed for them, and we make the following extract from his report to Messrs. Warner & Co.

"I take pleasure in testifying that your Quinine Pills are practically just what they claim to be, whether judging by analytical tests or by the therapeutic effects obtained from them." A. B. LYONS, M. D.,
Analytical Chemist.

Messrs. Warner & Co., make it their object to prepare pure and reliable medicines for the market, and their preparations will be found to be as they are represented in every instance.

